# HIV-1 A1 Subtype Epidemic in Italy Originated from Africa and Eastern Europe and Shows a High Frequency of Transmission Chains Involving Intravenous Drug Users

**DOI:** 10.1371/journal.pone.0146097

**Published:** 2016-01-11

**Authors:** Alessia Lai, Giorgio Bozzi, Marco Franzetti, Francesca Binda, Francesco R. Simonetti, Andrea De Luca, Valeria Micheli, Paola Meraviglia, Patrizia Bagnarelli, Antonio Di Biagio, Laura Monno, Francesco Saladini, Maurizio Zazzi, Gianguglielmo Zehender, Massimo Ciccozzi, Claudia Balotta

**Affiliations:** 1 Department of Biomedical and Clinical Sciences ‘L. Sacco’, Infectious Diseases and Immunopathology Section, ‘L. Sacco’ Hospital, University of Milan, Milan, Italy; 2 Division of Infectious Diseases, Siena University Hospital, Siena, Italy; 3 Laboratory of Microbiology, ‘L. Sacco’ Hospital, Milan, Italy; 4 2nd Division of Infectious Diseases, ‘L. Sacco’ Hospital, Milan, Italy; 5 Department of Biomedical Science, Section of Microbiology, Laboratory of Virology, University Politecnica delle Marche, Ancona, Italy; 6 Infectious Diseases Clinic, San Martino Hospital, Genoa, Italy; 7 Division of Infectious Disease, University of Bari, Bari, Italy; 8 Department of Medical Biotechnology, University of Siena, Siena, Italy; 9 Epidemiology Unit, Department of Infectious, Parasite and Immune-Mediated Diseases, Italian Institute of Health, Rome, Italy; Istituto Nazionale Tumori, ITALY

## Abstract

**Background:**

Subtype A accounts for only 12% of HIV-1 infections worldwide but predominates in Russia and Former Soviet Union countries of Eastern Europe. After an early propagation via heterosexual contacts, this variant spread explosively among intravenous drug users. A distinct A1 variant predominates in Greece and Albania, which penetrated directly from Africa. Clade A1 accounts for 12.5% of non-B subtypes in Italy, being the most frequent after F1 subtype.

**Aim:**

Aim of this study was to investigate the circulation of A1 subtype in Italy and trace its origin and diffusion through phylogenetic and phylodynamic approaches.

**Results:**

The phylogenetic analysis of 113 A1 *pol* sequences included in the Italian ARCA database, indicated that 71 patients (62.8%) clustered within 5 clades. A higher probability to be detected in clusters was found for patients from Eastern Europe and Italy (88.9% and 60.4%, respectively) compared to those from Africa (20%) (*p <* .*001*). Higher proportions of clustering sequences were found in intravenous drug users with respect to heterosexuals (85.7% *vs*. 59.3%, *p =* .*056*) and in women with respect to men (81.4% *vs*. 53.2%, *p <* .*006*). Subtype A1 dated phylogeny indicated an East African origin around 1961. Phylogeographical reconstruction highlighted 3 significant groups. One involved East European and some Italian variants, the second encompassed some Italian and African strains, the latter included the majority of viruses carried by African and Italian subjects and all viral sequences from Albania and Greece.

**Conclusions:**

Subtype A1 originated in Central Africa and spread among East European countries in 1982. It entered Italy through three introduction events: directly from East Africa, from Albania and Greece, and from the area encompassing Moldavia and Ukraine. As in previously documented A1 epidemics of East European countries, HIV-1 A1 subtype spread in Italy in part through intravenous drug users. However, Eastern European women contributed to the penetration of such variant, probably through sex work.

## Background

The evolution of HIV-1 subtype A, similar to subtype F, gave rise to different sub-subtypes (A1, A2, A3, A4) [1, 2], defined as sub-clusters and formed by distinct lineages highly related to the parental clade. The genetic distance between these subclades is about half of that between subtypes.

Subtype A is responsible of about 80% of the HIV-1 epidemic in Former Soviet Union (FSU) countries [3–5]. Evolving from a Central African ancestor, this variant initially spread through heterosexual (HE) contact in Southern Ukraine. Subtype A then begun to spread dramatically among injective drug users (IDUs) in the port city of Odessa around 1995. During the following years, the HIV-1 epidemic sustained by subtype A among IDU (labeled *A*_*fsu*_ or *IDU-A*) propagated through other FSU countries such as Russia and Estonia [6]. The explosive spread of this clade within the population of FSU IDUs was due to effective transmission networks taking place in the favorable setting of the massive socio-economic crisis which followed the fall of the Soviet Union Republics [7]. In the largely populated city of St. Petersbourg the proportion of *A*_*fsu*_ infections increased from 37% in the 1997–2004 period to 91% in 2006 of HIV-1 infections [6].

A feature of the IDU-A variant is a high homogeneity of viral sequences, probably due to the high transmission rates among IDUs following single introduction events in distinct geographic areas and the recent spread of the epidemic [8].

After its explosive diffusion in Russia, subtype A spread in neighboring countries [9–12]. In Bulgaria, subtype A was isolated only in a single individual before 1995, however, it accounted for 27% infections in further years [13], suggesting a late introduction through multiple events.

The prevalence of subtype A is approximately 2% in Western and Central Europe, however this variant has established extensive epidemics in some Mediterranean countries, such as Albania, Cyprus and Greece. In Greece, clade A1 is the most common non-B subtype (20.6%), rising from a 6% prevalence in 1984 to 42% in 2004 [14, 15]. The introduction of this subtype in Greece dates back to the first epidemic phase in the country (time of the most recent common ancestor, tMRCA, 1978), probably originating from Central Africa [16]. Differently from other European countries, subtype A is the most frequent in long-dated residents compared with subtype B, and was involved in sexual transmission risk groups more lately [14]. Similarly, the clade A epidemic in Albania probably arose from Greece, Albanian and Greek sequences being more related to African ones than to Eastern European ones [17].

Clade A is a parental subtype in most of known circulating recombinant forms (CRFs), particularly in the most prevalent ones. These CRFs are estimated to sustain 27% HIV-1 infections globally [18], especially CRF02_AG in Western Africa [19], CRF01_AE in Thailand [20] and CRF03_AB in the FSU. The high prevalence of co-circulating subtype A and B in Eastern Europe represented the background for the origin of CRF03_AB in Southern Ukraine, giving rise to an outbreak through IDUs in the city of Kaliningrad in 1996 [21].

Due to migration fluxes from Africa, FSU and South America, non-B subtype circulation is increasing in Italy as well as in all Western countries of Europe. The overall prevalence of infections due to non-B clade in Italy is 11.4% having raised from 2.6% to 18.9% over three decades. Among these, subtype A is the second in prevalence (12.7%) after clade F1 (23.7%) [22]. The aim of this study was to investigate the features of A1 subtype circulation in Italy and trace its origin and diffusion through phylogenetic and phylodynamic approaches.

## Patients and Methods

### Study population

We studied 113 individuals carrying HIV-1 A1 subtype. Patients were sampled from 1999 through 2011. Subjects signed an informed consent to have their anonymized data stored on a central server of the ARCA database (www.dbarca.net) and used for research studies. Authors working in the clinical setting interacted with some of the patients included in the study as part of their own routine HIV care. No additional visits were scheduled, apart from those regularly planned for HIV monitoring, according to Italian national guidelines. ARCA is an observational HIV cohort approved by the Regional Ethical Committee of Tuscany (Comitato Etico Area Vasta Toscana Sudest). HIV-1 protease (PR) and partial reverse transcriptase (RT) sequences were generated by local centers for routine drug resistance testing at diagnosis or prior to the start of antiretroviral therapy or at treatment failure. Epidemiological data (gender, risk category, country of origin, date of diagnosis and age) were collected by physicians from patient medical records and then included in the databases together with virological, immunological and treatment information. Only the earliest available HIV-1 genotype was considered for each patient. The study was conducted in accordance with the 1964 Declaration of Helsinki and the ethical standards of the Italian Ministry of Health.

### Phylogenetic dataset

The analysis of epidemiological clusters was performed on the starting dataset of 113 HIV-1 A1 subtypes.

The evolutionary population dynamics was estimated in the dataset of Italian patients (n = 53).

A reference set of 229 subtype A1 *pol* gene sequences was obtained from the Los Alamos HIV Sequence Database (www.hiv.lanl.gov). Viral sequences were selected according to the following inclusion criteria: i) sequences had already been published in peer-reviewed journals; ii) the subtype assignment of each sequences classified as non-recombinant was certain; iii) the city/state of origin and the year of sampling were known and clearly established in the original publication. The final reference set includes subtype A1 sequences of Central Africa (n = 22; Democratic Republic of Congo = 13, Central African Republic = 3, Gabon = 6), East Africa (n = 52; Uganda = 17, Tanzania = 16, Kenya = 19), Estonia (n = 9), Latvia (n = 19), Lithuania (n = 5), Moldavia (n = 5), Ukraine (n = 20), Byelorussia (n = 10), Russia (n = 20), Kazakhstan (n = 6), Uzbekistan (n = 7), Greece (n = 9), Albania (n = 8). The accession number list of phylogeographic references is provided in S1 Table Thirty seven randomized sequences belonging to patients with established Italian citizenship present in a starting dataset were included. The phylogeographic dataset of 229 isolates was used to evaluate the migration pattern of HIV-1 subtype A1 circulation. The phylogenetic signal was evaluated on the phylogeographic dataset.

### Alignment and phylodynamic analysis

The sequences were manually aligned using Bioedit software (v7.2.5; http://www.mbio.ncsu.edu/bioedit/bioedit.html). All sites of major antiretroviral drug resistance mutations in the protease and reverse transcriptase regions, identified through the last updated Surveillance Drug Resistance Mutations (SDRM) (http://hivdb.stanford.edu/cgi-bin/AgMutPrev.cgi) and International AIDS Society (IAS) (http://www.iasusa.org/guidelines/index.html) lists for naïve and treated patients respectively, were removed to avoid bias due to convergence related to drug resistance. The final alignment encompassed 834 nucleotides.

The Bayesian phylogenetic tree was reconstructed by means of both MrBayes and Beast using the general time reversible (GTR)+ gamma distribution (G)+ proportion of invariable sites (I) model, which was selected using the Modeltest v. 3.7 program. A Markov Chain Monte Carlo (MCMC) search was made for 10x10^6^ generations using tree sampling every 100th generation and a burn-in fraction of 50%. Statistical support for specific clades was obtained by calculating the posterior probability (pp) of each monophyletic clade, and a posterior consensus tree was generated after a 50% burn-in. Clades with a pp of 1 were considered epidemiologically related clusters.

Dated trees, evolutionary rates and population growth were co-estimated by using Beast v.1.8.0 [23] software (http://beast.bio.ed.ac.uk) using the previous model. Different coalescent priors (constant population size, exponential growth, logistic growth and Bayesian skyline plot) were tested using both strict and relaxed molecular clock models. Different clock models were compared by calculating the Bayes Factor (BF) [15]. The difference (in log_e_ space) of marginal likelihood between any two models is log_e_ BF. Evidence against the null model (i.e. the one with lower marginal likelihood) was indicated by: 6>(2 log_e_ BF)>2 (positive), 10>(2 log_e_ BF)>6 (strong) and (2 log_e_ BF)>10 (very strong) [16]. BF calculations were performed with Tracer v.1.5 software (http://beast.bio.ed.ac.uk/Tracer). The BF analysis showed that the relaxed lognormal molecular clock fitted the data better than the strict clock model (2lnBF = 114.5). Chains were run for 50 million generations with sampling every 5,000 generations. The results were visualized in Tracer v.1.5 and uncertainty in the estimates was indicated by 95% highest probability density (HPD) intervals. Convergence was assessed based on the ESS (effective-sample size) value and only parameter estimates with ESS>300 were accepted. The maximum clade credibility (MCC) tree was then selected from the posterior tree distribution using TreeAnnotator v.1.8.0 available in the BEAST software package. Final trees were visualized and manipulated with FigTree v.1.4.0 (http://tree.bio.ed.ac.uk/software/figtree/).

### Likelihood mapping analysis

In order to obtain an overall feature of the phylogenetic signal present in the HIV-1 A1 sequences, we made a likelihood-mapping analysis of 10,000 random quartets generated using TreePuzzle (Schmidt et al., 2002). A likelihood map consists of an equilateral triangle: each dot within the triangle represents the likelihoods of the three possible unrooted trees for a set of four sequences (quartets), randomly selected from the dataset. The dots close to the corners or at the sides respectively represent tree-like (fully resolved phylogenies in which one tree is clearly better than the others) or network-like phylogenetic signals (three regions for which it is not possible to decide between two topologies). The central area of the map represents a star-like signal (the region where the star tree is the optimal tree).

### Phylogeographic analysis

Analyses were performed using Bayesian Skyline coalescent tree prior with a relaxed clock (uncorrelated Lognormal model). Chains were run for 100 million generations with sampling every 10,000. The results were visualized using Tracer v.1.5. The ESS value for each parameter was >300, indicating sufficient mixing of the Markov chain. Rates with a BF of >3 were considered well supported and formed the migration pathway. The MCC tree was selected from the posterior tree distribution after a 10% burn-in using the TreeAnnotator program, version 1.8.0. The final tree was visualized using FigTree version 1.4.0. To provide a spatial projection, the migration routes indicated by the tree were visualized using Google Earth (http://earth.google.com) and the SPREAD program (available at http://www.kuleuven.ac.be/aidslab/phylogeography/SPREAD.html).

### Viral gene flow analysis

The MacClade program version 4 (Sinauer Associates, Sunderland, MA) was used to test viral gene out/in using a modified version of the Slatkin and Maddison test [24]. A one-character data matrix was obtained from the original dataset by assigning a one-letter code, indicating the geographical origin, to each taxon in the tree. The putative origin of each ancestral sequence (i.e., internal node) in the tree was then inferred by finding the most parsimonious reconstruction (MPR) of the ancestral character using either the ACCTRAN or DELTRAN option. The final tree length, that is the number of observed viral gene flow events in the genealogy, is computed and compared to the tree-length distribution of 10,000 trees obtained by random joining-splitting (null distribution). Observed genealogies significantly shorter than random trees indicate the presence of subdivided populations with restricted gene flow. The viral gene flow (migrations) was traced with the ‘State changes and stasis tool’ (MacClade software), which counts the number of changes in a tree for each pairwise character state. Gene flow was also calculated for the null distribution to assess whether the gene flow events observed in the actual tree were significantly higher (>95%) or lower (<95%) than the values in the null distribution at the *p =* .*05* level.

### Statistical methods

Categorical variables were summarized as frequencies and comparisons between groups were performed using the χ2 or the Fisher exact test. For all analyses an α error of 5% was considered. The crude and Mantel–Haenszel-adjusted odds ratios of inclusion in clusters with 95% CIs were calculated in univariate and multivariate analyses. Analyses were performed using the SPSS software package (v.21.0, SPSS Inc. Chicago, IL, USA).

## Results

### Patient characteristics

One-hundred thirteen HIV-1 A1 infected patients with sequence data spanning a 13-year period (Table 1) were included. Italians, East Europeans, Africans, South Americans and Asians accounted for 46.9% (n = 53), 15.9% (n = 18), 8.8% (n = 10), 1.8% (n = 2) and 0.1% (n = 1) of patients with known country of origin, respectively. Known risk factors were distributed as follows: 64.3% (n = 27) HEs, 19% (n = 8) men having sex with men and 16.7% (n = 7) IDUs. Males accounted for 59% of patients (n = 62). Median age was 35 years (range: 24–44 years). Naïve patients accounted for 21.2% (n = 24); only two patients harbored resistant strains (8.3%) and only mutations selected by Protease Inhibitors (PI) were present.

**Table 1:** Characteristics of 113 patients carrying subtype A1.

Patient characteristics	All patients	Patients within clusters	Patient outside clusters	*p*
**ORIGIN % (n)**				
Italy	46.9 (53)	60.4 (32)	39.6 (21)	
East Europe	15.9 (18)	88.9 (16)	11.1 (2)	
Africa	8.8 (10)	20.0 (2)	80.0 (8)	*p <* .*001*
South America	1.8 (2)	100 (2)	0.0 (0)	
Asia	0.1 (1)	100 (1)	0.0 (0)	
Unknown	26.5 (29)	96.6 (28)	3,4 (1)	
**RISK FACTOR % (n)**				
HEs	64,3 (27)	59.3 (16)	40.7 (11)	
MSM	19.0 (8)	25.0 (2)	75.0 (6)	*p =* .*056*
IDUs	16,7 (7)	85.7 (6)	14.3 (1)	
**GENDER % (n)**				
Male	59.0 (62)	53.2 (33)	46.8 (29)	*p <* .*006*
Female	38.2 (97)	81.4 (35)	18.6 (62)	
**AGE (median years)**	35	33	39	

### Associations between subtype A and patients characteristics

The phylogenetic trees were constructed on 113 individuals carrying HIV-1 A1 subtype. No differences were observed in the topology of trees obtained using MrBayes or BEAST programs. The BEAST tree of HIV-1 A1 sequences is shown in S1 Fig. Based on the latter tree we identified 5 highly supported clades (with posterior probability, pp, equal to 1) (S1 Fig) that included 71 patients (62.8%). A large network (#5) encompassed 59 isolates (83.1% of clustering sequences), while the remaining four included three patients each. The major clade involved all patients from Eastern Europe and included 47.2% of Italians (n = 25). Noteworthy, female gender accounted for 54.2% (n = 32) in cluster #5. Differently to the other networks, cluster #4 involved only Italian subjects. Clusters #1 and #4 included only treated subjects. None of the clusters included exclusively subjects with the same gender.

The overall epidemiological characteristics and those of patients included or not within epidemiological networks are described in Table 1.

Patients from Eastern Europe and Italy had a higher probability to be detected in clusters (n = 16, 88.9% and n = 32, 60.4%, respectively) compared to those from Africa (n = 2, 20%) (*p <* .*001*). The proportion of clustering sequences was slightly higher in IDUs (n = 6, 85.7%) and HEs (n = 16, 59.3%) compared to MSM (n = 2, 25%) (*p =* .*056*). Women carried a higher proportion of clustering sequences (n = 35, 81.4%) compared to men (n = 33, 53.2%) (*p <* .*006*). Among clustering females with known risk factor, 80% (n = 8) were HEs and 20% IDUs. Seven of HE females were from East Europe (87.5%).

Male gender was predominant both in total population and in Italian patients within clusters (n = 38, 71.7% *vs*. n = 19, 59.4%, respectively) (*p <* .*001*), whereas female gender was prevalent among patients from Eastern Europe (n = 13, 16.7% *vs*. n = 15, 18.7%) (*p =* .*029*). No difference was observed in the distribution of naïve subjects inside or outside clusters, however 19 (79%) naïve individuals were present in the epidemiological chains. Patients involved in such chains were significantly younger (33 years, range: 28.5–40 years) than patients outside clusters (39 years, range: 34–53.5 years) *(p =* .*003)*.

### Phylodynamic analysis

**Fig 1:**
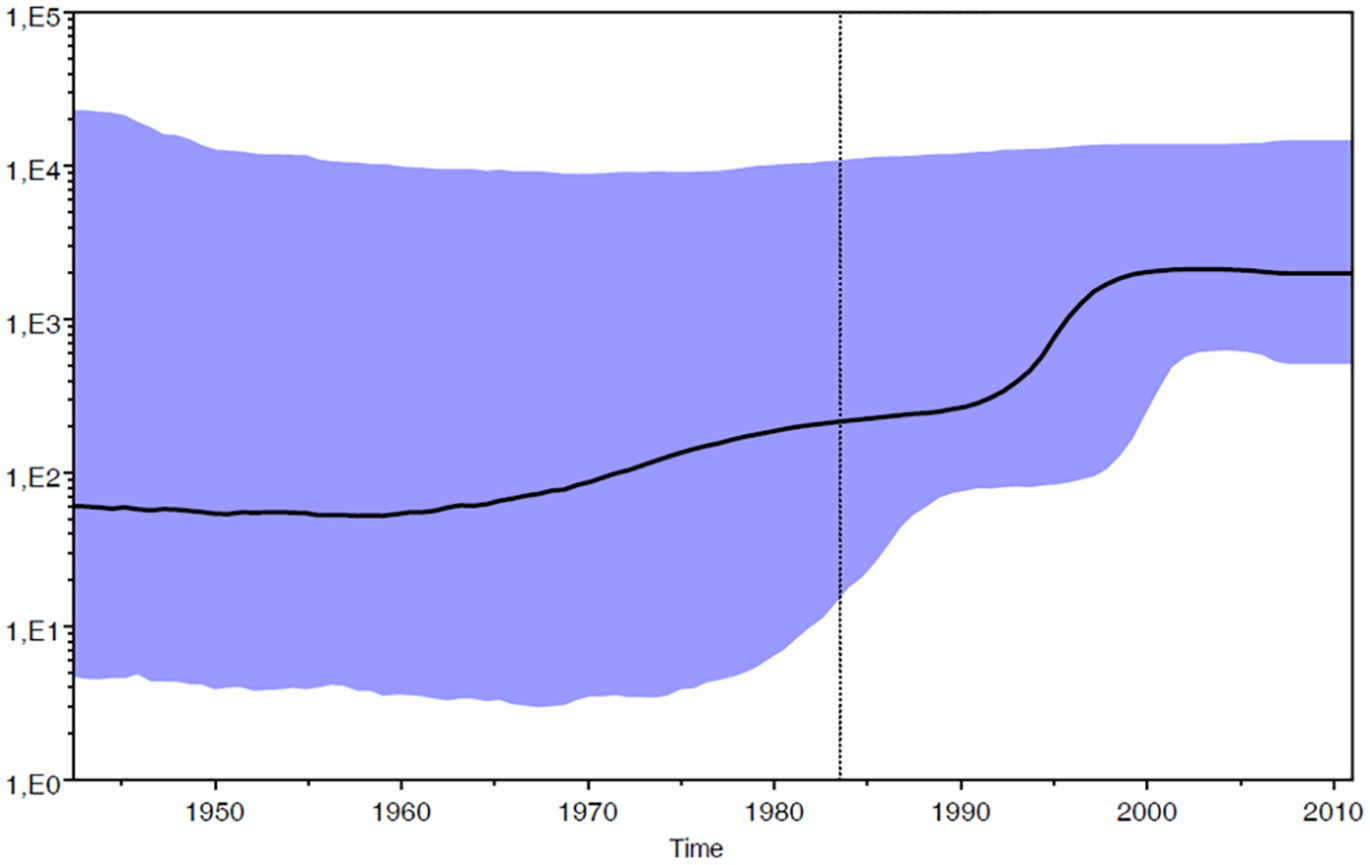
Skyline plot obtained by analyzing the data set of Italian patients (n = 53). Ordinate: the number of effective infections at time t (Ne(t)); abscissa: time (in years before the present). The thick solid line represents the median value and the grey area the 95% HPD of the Ne(t) estimates. The vertical lines indicate the 95% lower HPD (dotted) and the mean tMRCA estimate (bold) of the tree root.

The phylodynamic analysis was conducted on the dataset of patients with certain Italian citizenship (n = 53) carrying HIV-1 A1 subtype. The Bayesian Skyline plot showed the changes in population size at different times from the root of the tree to the time of the most recent isolates (year 2011). The mean substitution rate for the analyzed dataset was 1.11 x 10^−3^ (95% HPD: 2.82 x 10^−4^–1.87 x 10^−3^). The effective number of infections grew exponentially approximately between 1990 and 1998 and reached a plateau around 2000, when the effective number of infections remained constant until the last calendar years (Fig 1).

### Phylogeographical reconstruction

The likelihood mapping analysis of the HIV1-A1 dataset of 229 isolates showed that the percentage of dots falling in the central area of the triangles was 4.2%, thus indicating a fully resolved phylogenetic signal (S2 Fig).

**Fig 2:**
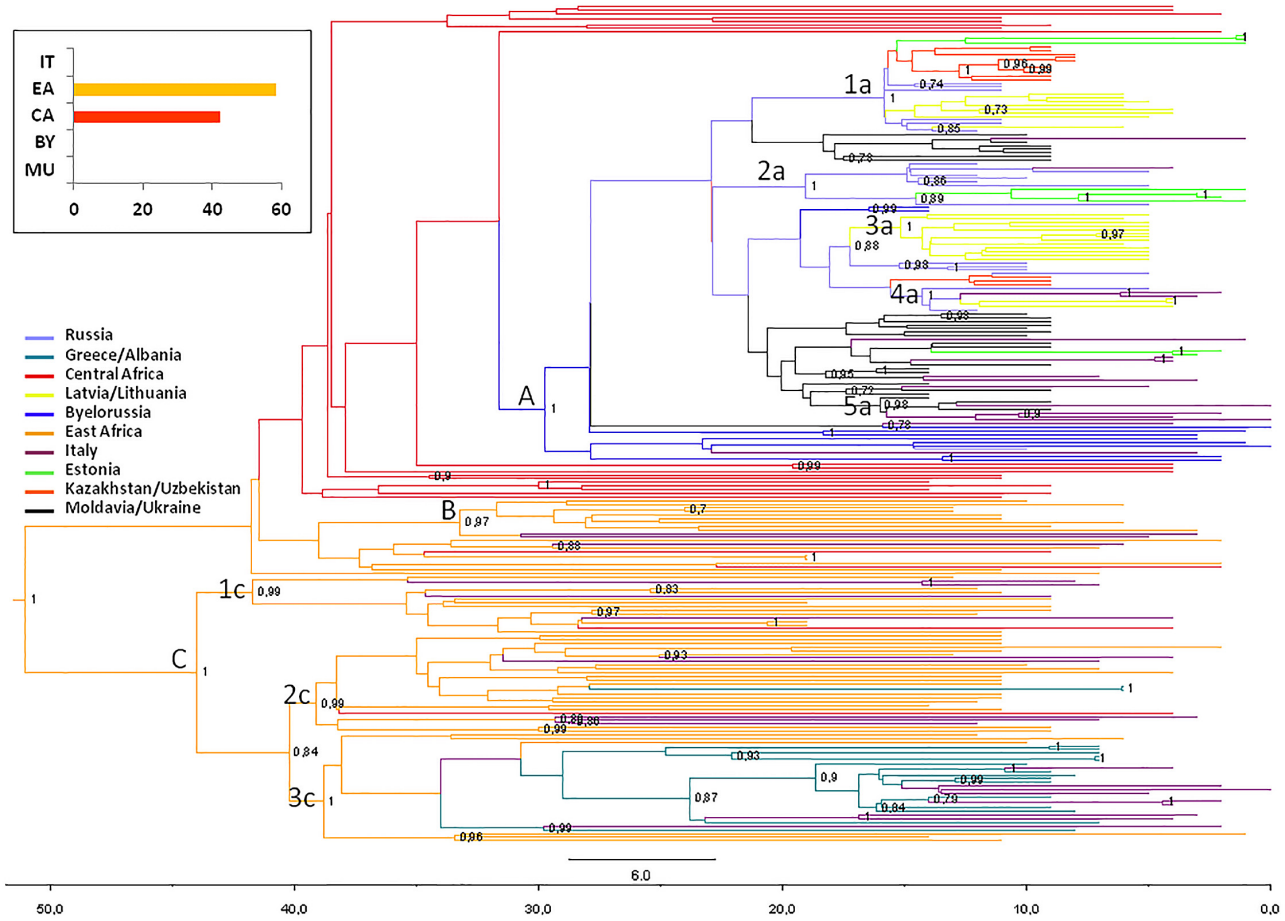
Bayesian phylogeographical tree of 229 HIV-1 A *pol* sequences with branches colored on the basis of the most probable location of the descendent nodes. The correspondences between the locations and colors are shown in the panel (left), and the posterior probabilities >0.8 are indicated on the internal nodes of the tree. The scale axis below the tree shows the number of years before the last sampling time.

The phylogeographic maximum clade credibility (MCC) tree, obtained grouping the 229 isolates within ten discrete groups on the basis of the sampling location (IT, Italy; CA, Central Africa; EA, East Africa; EE, Estonia; LL, Latvia/Lithuania; MU, Moldavia/Ukraine; BY, Byelorussia; RU, Russia; KU, Kazakhstan/Uzbekistan; GA, Greece/Albania) is shown in Fig 2.

The most probable location of the root of the tree was East Africa, supported by a state posterior probability (spp) significantly higher than that of the other locations (spp = 0.58 *vs*. spp = 0.42 for Central Africa, the second most probable location of the root). The date of the most recent common ancestor (MRCA), corresponding to the East African root, was estimated to be 51 years (95% HPD = 42.1–69.4 years), corresponding to the year 1960.

Phylogeographical reconstruction highlighted 3 highly supported groups (A, pp = 1; B, pp = 0.97; and C, pp = 1). Group A involved all sequences from Eastern Europe and the most probable location is Byelorussia (spp = 0.76). Sixteen Italian sequences were interspersed within this group. The mean tMRCA of the A group was 29.7 years, (95% HPD = 22–35.7 years) corresponding to 1981. In this group five clusters can be identified (1a, pp = 1; 2a, pp = 1; 3a, pp = 1; 4a, pp = 1; and 5a, pp = 0.98). The first cluster (1a) included 27 isolates (10 from Kazakhstan/Uzbekistan, 3 from Estonia, 6 from Russia and 8 from Latvia/Lithuania); cluster 2a involved 12 strains (7 from Russia, 4 from Estonia and 1 from Italy); cluster 3a contained only sequences from Latvia/Lithuania (n = 13); cluster 4a contained 7 isolates (3 from Latvia/Lithuania, 2 from Russia and 2 from Italy); cluster 5a included 7 isolates (4 from Italy and 3 from Moldavia/Ukraine). The mean tMRCA of these clusters range from 15–19 years (95% HPD = 12–20 years).

Group B involved 9 African sequences and two Italian strains and the most probable location is East Africa (spp = 1). The mean tMRCA of B group was 41.8 years (95% HPD = 32.8–42.7 years).

Group C involved the majority of African sequences (spp = 1 for East Africa), 18 Italian sequences, all strains from Greece/Albania and 2 Central African sequences. The mean tMRCA of group C was 44 years, (95% HPD = 36.1–47.8 years) corresponding to 1967. In this group three clusters were identified (1c, pp = 0.99; 2c, pp = 0.99; and 3c, pp = 1). The first cluster (1c) included 16 isolates (11 from East Africa, 4 from Italy and 1 from Central Africa); the cluster 2c involved 27 strains (19 from East Africa, 5 from Italy and 1 from Central Africa); cluster 3c contained 30 sequences (6 from East Africa, 15 from Greece/Albania and 9 from Italy). The mean tMRCA of these clusters range from 38–42 years (95% HPD = 32.2–44 years). tMRCA values, confidence intervals and the locations of the main groups and clusters are listed in Table 2.

**Table 2:** tMRCAs, credibility intervals (95%HPD) and location of the main groups (A, B, C) and clades (1a, 2a, 3a, 4a, 5a, 1c, 2c, 3c) on the basis of the dataset of 229 HIV-1 subtype A1 isolates.

Variable	tMRCA	95% HPD	Location
Root	51	42.1–69.4	East Africa
A	30	21.9–35.7	Byelorussia
1a	16	15.4–20	Russia
2a	19	14.2–18.4	Russia
3a	15	13.8–18.6	Latvia/Lithuania
4a	16	Dec-17	Russia
5a	16	14.4–18.3	Moldavia/Ukraine
B	42	32.8–42.7	East Africa
C	44	36.1–47.8	Central Africa
1c	42	32.8–42.7	East Africa
2c	39	34–44	East Africa
3c	39	32.2–42.4	East Africa

tMRCA: time of the most recent common ancestor; HPD: highest posterior density.

### Significant linkages

Bayesian phylogeographic analysis of 229 HIV-1 A1 isolates showed eleven non-zero rates between ten discrete locations (Italy, East Africa, Central Africa, Estonia, Latvia/Lithuania, Moldavia/Ukraine, Byelorussia, Russia, Kazakhstan/Uzbekistan and Greece/Albania). Significant linkage (BF>3.0) was found between Byelorussia and Moldavia/Ukraine (BF = 6), Central Africa (BF = 7.1) and Russia (BF = 3.2). Russia was significantly linked with Estonia (BF = 17), Kazakhstan/Uzbekistan (BF = 20), Latvia/Lithuania (BF = 32778.4) andMoldavia/Ukraine (BF = 26.8). Italy was significantly linked with three locations: East Africa (BF = 297.1), Greece/Albania and Moldavia/Ukraine (BF = 32778.4 for both locations). Central Africa and East Africa are linked with a BF = 5660.

**Fig 3:**
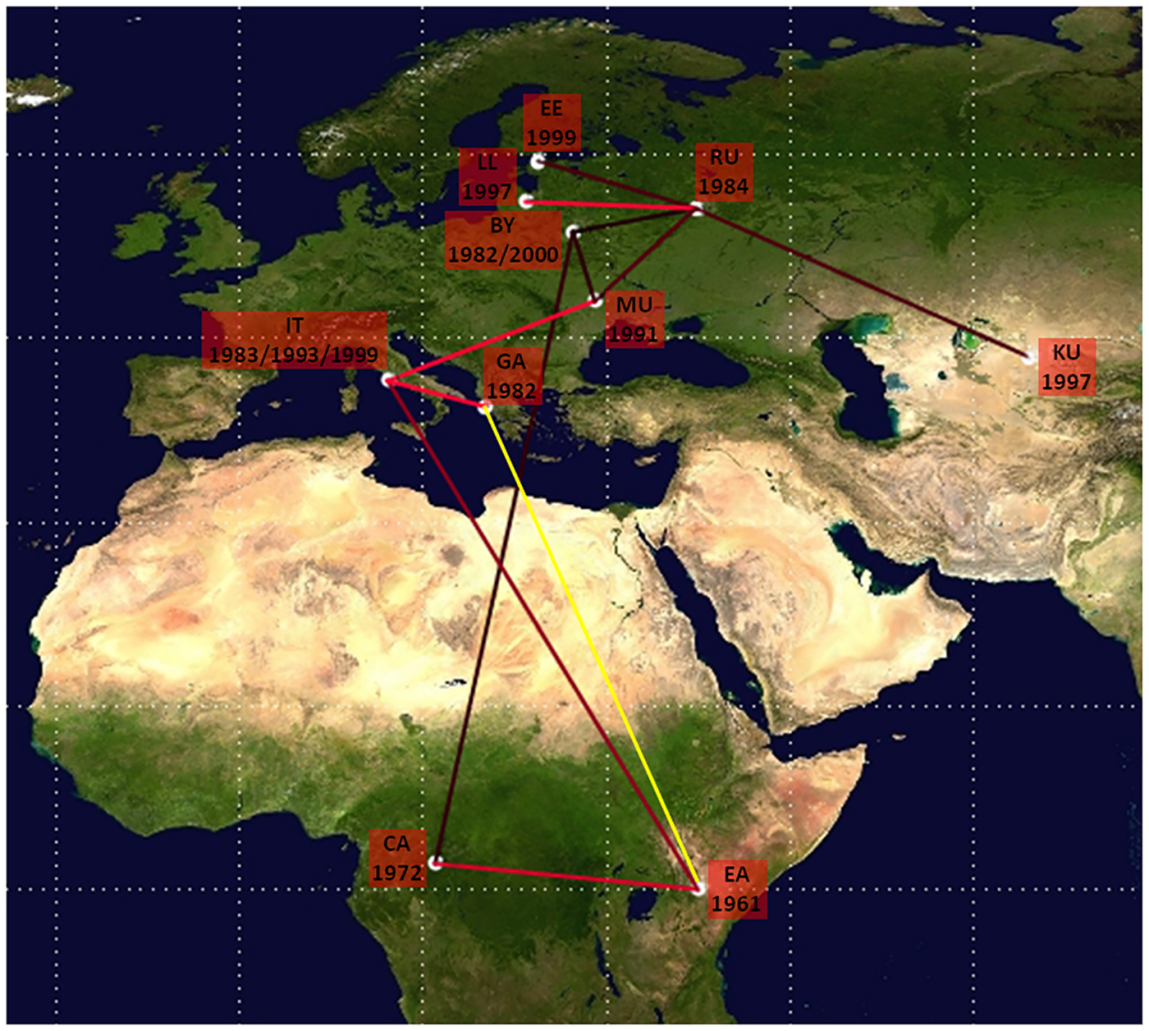
Significant non-zero HIV-1 A1 migration rates worldwide. Only the rates supported by a BF of >3 are shown in red, yellow indicates the probable rate. The relative strength of the statistical support is indicated by the color of the lines (from dark red, *id est* weak to light red *id est* strong). The map was reconstructed using SPREAD program. This figure is similar but not identical to the original image, and is therefore for illustrative purposes only.

The pathways of HIV-1 A1 dispersion are showed in Fig 3. HIV-1 A1 subtype originated in East Africa around 1961 and spread to Central Africa in 1972. Subsequently, the virus spread in East Europe starting from Byelorussia where the virus was introduced in 1982 from Central Africa. The virus spread to the Kazakhstan/Uzbekistan from Russia around 1996.

The HIV-1 A1 subtype reached Italy primarily from East Africa (year 1983) and subsequently from Greece/Albania and Moldavia/Ukraine around 1993–1999. A probable link related East Africa and Greece/Albania where the virus was introduced in 1983, even though a not significant BF could be observed.

### Migration rates and fluxes

**Fig 4:**
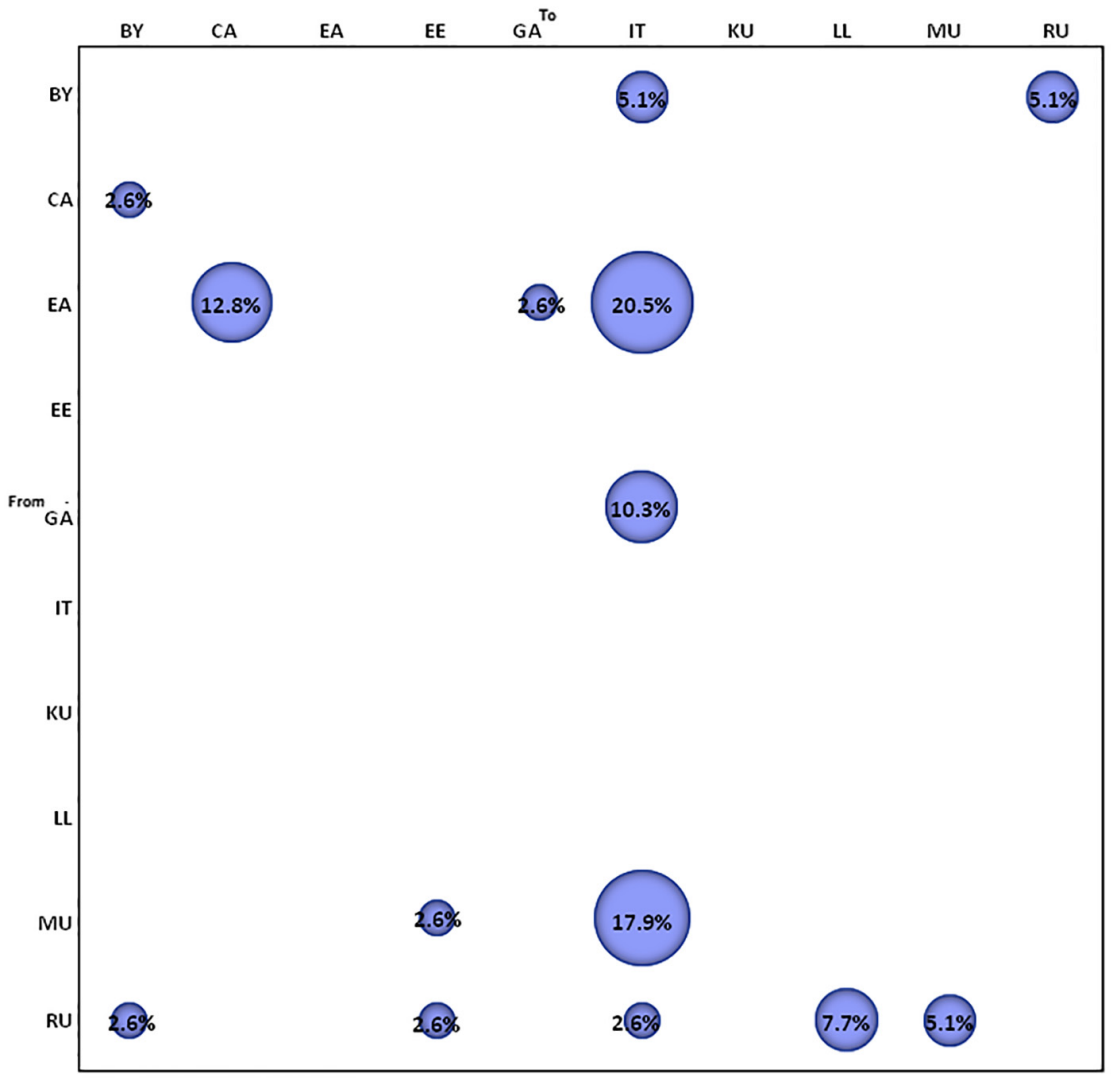
Migration pattern of HIV-1 subtype A1 circulation based on phylogeographic dataset. The bubblegram shows the frequency of gene flow (migrations) to/from different geographic areas. The surface of each circle is proportional to the percentage of observed migrations in the Maximum Likelihood genealogy. Migrations were inferred with a modified version of the Slatkin and Maddison algorithm. BY, Byelorussia; CA, Central Africa; EA, East Africa; EE, Estonia; GA, Greece/Albania; IT, Italy; KU, Kazakhstan/Uzbekistan; LL, Latvia/Lithuania; MU, Moldavia/Ukraine; RU, Russia.

The migration pattern of HIV-1 subtype A1 circulation was calculated using the phylogeographic dataset of 229 isolates. The gene flow (migration) between different geographic areas was investigated with a modified version of the Slatkin and Maddison method. The null hypothesis of panmixia (i.e. no population subdivision or complete intermixing of sequences from different geographical areas) was tested using a bubblegram. The migration flow among the five locations was then estimated as observed migration in the genealogy (Fig 4). The null hypothesis of panmixia was ejected by the randomization test (*p =* .*0001*) for all the countries in the bubblegram. ACCTRAN resolving option was used for uncertainties present in the resulting tree.

HIV-1 A1 gene flows were observed from East Africa to Central Africa (12.8%), Greece/Albania (2.6%) and Italy (20.5%). Italy received migrations also from Byelorussia (5.1%), Greece/Albania (10.3%), Moldavia/Ukraine (17.9%) and Russia (2.6%).

The gene flow from Central Africa to Byelorussia, from Byelorussia to Russia and from Moldavia/Ukraine to Estonia accounted for 2.6%, 5.1% and 2.6% of the observed migrations, respectively (Fig 4). HIV-1 subtype A1 gene flow indicated Russia as the epicenter of HIV-1 A1 epidemic in East Europe, branching out to Byelorussia (2.6%), Moldavia/Ukraine (5.1%) and Latvia/Lithuania (7.7%) (Fig 4).

## Discussion

The prevalence of non-B subtypes has increased over time in Italy as well as in Europe and this rise can be attributed mainly to an increasing frequency of F1, C, CRF02_AG and A1 subtypes [22].

The present study is the first detailed investigation of the HIV-1 A1 molecular epidemiology in Italy using a national database and a Bayesian phylogenetic approach.

An extraordinary proportion of variants were associated to A1 epidemiological networks (more than 62% of studied sequences). We observed a relevant role of heterosexual contacts and injecting drug use. Italian male heterosexuals and IDUs predominated while foreign-born patients were mainly heterosexual females from East Europe. The high frequency of Italian heterosexual males and Eastern Europe females within epidemiological networks indicated that sexual contacts have led to a relevant circulation of subtype A1 in Italy. Only one cluster involving patients with Italian citizenship was identified, while the majority of Italian strains were intermixed within East European patients. Additionally, despite the reported decrease of infections due to injecting drug usage (http://www.iss.it/binary/ccoa/cont/ONLINE_6_COA_2014.pdf), the circulation of A1 subtype partially remains related to this modality of infection as in previously documented A1 epidemics of East European countries [21]. In contrast with previous published data regarding other non-B subtypes circulating in Italy, such as subtype C and F1 which mobility is associated with men having sex with men, the A1 subtype remains related to heterosexuals and IDUs [25, 26].

The present study also investigated the HIV-1 A1 epidemics in Italy and worldwide, with the aim to establish the origin of the A1 variant circulating in Italy as well as in the European and African context. The present phylogenetic analysis showed that subtype A1 originated, as expected, in East Africa (where subtype A is the most prevalent clade) with a recent epidemic, originating around 1961 [27].

Global subtype A1 dispersion showed a strong geographic compartmentalization in many clusters. Dispersal routes of subtype A1 from Africa to Europe were probably associated with immigration from Africa while viral mobility within Eastern Europe is probably due to connections among IDUs. After its origin in East Africa subtype A1 migrated in Central Africa; both variants from East and Central Africa likely originated the epidemic in Europe. The spread among East European countries started from 1982, corresponding to the identification of the first outbreaks in countries formerly part of Soviet Union as reported by literature [7].

In general, several subtype A migration pathways exist among European countries which are mainly associated with heterosexuals and IDUs in Eastern Europe. Greece and Albania are a consistent exception due to the establishment of a large HIV-1 subtype A local epidemic sustaining 20.6% and 56.1% of infections, respectively [13, 14]. In agreement with data of Paraskevis et al. [14], our findings showed a monophyletic origin of subtype A epidemic in Greece and Albania and confirmed its origin in East Africa (Kenya and Tanzania) with a date of origin around 1983.

Phylogeographical reconstruction suggests that A1 clade entered Italy through three distinct introduction events: an early first getaway took place directly from East Africa, where subtype A has a high prevalence, while two additional events occurred from the Southern Balkan Peninsula and the area encompassing Moldavia and Ukraine. This phenomenon could be related to Eastern European women who contributed to the penetration of such variant probably through sex work as demonstrated by the high proportion of women in clusters. It has been documented that, during the past 10 years, Albanian female sex workers traveled to other Balkan countries and to Western Europe, especially Italy and Greece. The very low percentage of African subjects within epidemiological networks suggests a minor role of the African A1 variant compared to the East European one in the circulation of this subtype in Italy. This is in agreement with the high degree of homogeneity of the virus populations carried by IDUs in the former Soviet Union countries which suggests that they shares a quite recent common ancestor, as a result of a single introduction. In contrast, HIV-1 A1 African sequences presented a high genetic variability, that increased in Democratic Republic of Congo in comparison to in West or East Africa [11, 16, 28].

The estimation of the demographic history of subtype A1 in Italy revealed that the epidemic growth during the 1990s. The stabilization of the epidemic around 2000 can be probably explained by the reduced number of infections related to injecting drug usage and the diffusion of combination antiretroviral therapy in infected IDUs.

Globally, we hypothesize three different coeval introduction events of the A1 variant from Africa into Europe; the former involving Byelorussia [29], the second Greece and the latter Italy. The migration fluxes confirmed the results of phylogeographic reconstruction giving a strong support to the link between East Africa and Greece.

Some possible limits and biases of the present study have to be mentioned. First, although our dataset includes sequences from a national database we cannot exclude the possibility that the study population is not truly representative of all HIV-1 A1 subtype cases in Italy. Another potential limit of our study is that we analyzed a portion of *pol* gene, rather than full genome sequences, which could underestimate recombinant strains that frequently include A1 subtype portions (such as CRF01_AE or CRF02_AG, very frequent in Italy). Nevertheless, phylogenetic and recombination analysis were performed to confirm the subtype assignment on sequences classified as A1 subtype in the ARCA database by also comparing these strains with other known CRFs. Additionally, the relative lack of country of origin and risk factor information for some patients could have weakened the strength of the observed associations.

In conclusion, this study provides novel information about the A1 subtype epidemic and its geographic origin, as well as the routes of transmission among patients living in Italy and carrying such variant. Due to its founder effect, subtype B predominates the HIV-1 Italian epidemic. However, the geographic position of Italy in the center of Mediterranean basin and the increase of population mobility in Europe, had the potential to modify the previous HIV-1 epidemiological structure. The findings in the present study contribute to the understanding of this changing pattern ad highlight the importance of an extensive and continuous monitoring of the spread of HIV-1 variants in Europe.

## Supporting Information

S1 Fig Bayesian tree of 113 HIV-1 A1 isolates obtained with Beast program. The 5 supported clusters are highlighted in red color. Node labels indicate posterior probability values (pp). Only values higher than 0.8 are shown.
(TIF)

S2 Fig Likelihood mapping of the HIV-1 A1 data sets of 229 nucleotide sequences obtain using TreePuzzle program. The three corners represent fully resolved tree topologies, id est the presence of a tree-like phylogenetic signal in the given data set.
(TIF)

S1 Table Accession numbers of used references. (DOCX)
