# Supplementary figures and images for: HIV-1 A1 Subtype Epidemic in Italy Originated from Africa and Eastern Europe and Shows a High Frequency of Transmission Chains Involving Intravenous Drug Users
Source: PLoS One. 2016 Jan 11;11(1):e0146097. doi: 10.1371/journal.pone.0146097 (PMC4709132; doi:10.1371/journal.pone.0146097)

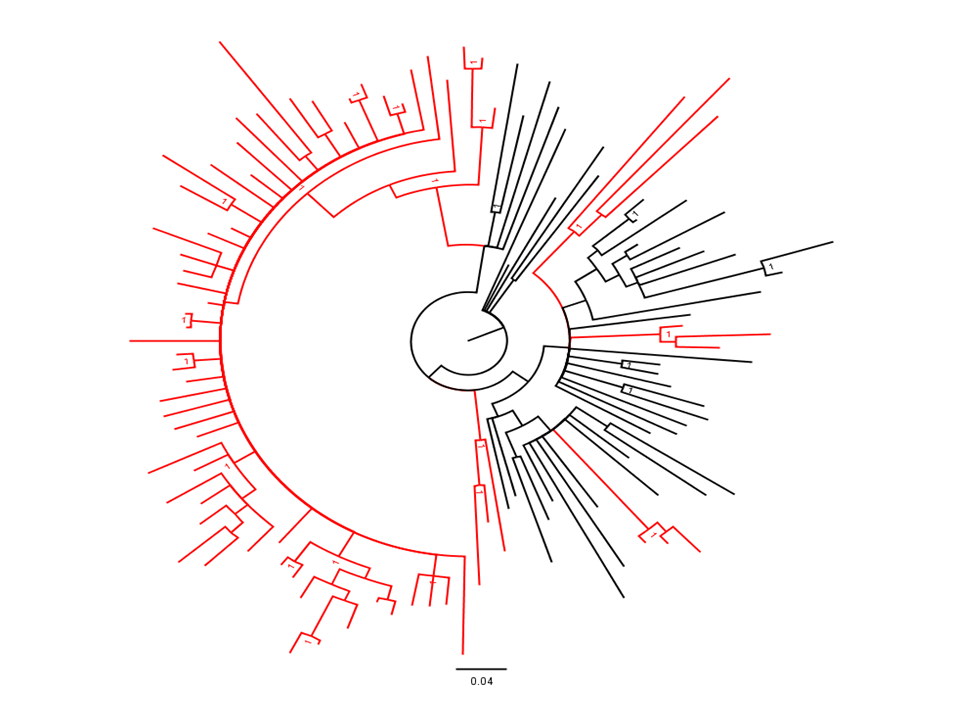

Supplement: S1 Fig — The 5 supported clusters are highlighted in red color. Node labels indicate posterior probability values (pp). Only values higher than 0.8 are shown. (TIF) [file pone.0146097.s001.tif]

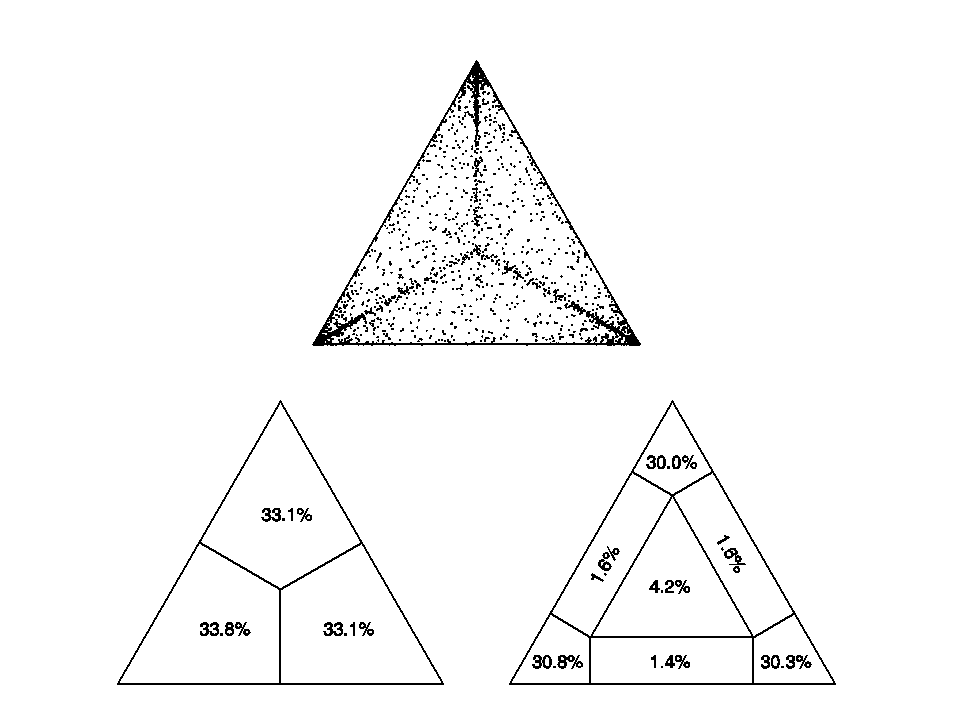

Supplement: S2 Fig — The three corners represent fully resolved tree topologies, id est the presence of a tree-like phylogenetic signal in the given data set. (TIF) [file pone.0146097.s002.tif]
