# Supplementary material for: HIV-1 A1 Subtype Epidemic in Italy Originated from Africa and Eastern Europe and Shows a High Frequency of Transmission Chains Involving Intravenous Drug Users
Source: PLoS One. 2016 Jan 11;11(1):e0146097. doi: 10.1371/journal.pone.0146097 (PMC4709132; doi:10.1371/journal.pone.0146097)
Supplement: S1 Table — (DOCX) [file pone.0146097.s003.docx]

S1 Table. Accession numbers of used references.

| **Democratic Republic of Congo=13** | AM000055 |
| --- | --- |
|  | AM000053 |
|  | AM000054 |
|  | FR666651 |
|  | FR666632 |
|  | FR666606 |
|  | FR666639 |
|  | FR666640 |
|  | FR666633 |
|  | FR666612 |
|  | AM041053 |
|  | AM041049 |
|  | AM041032 |
| **Central African Republic=3** | JF803979 |
|  | JF803996 |
|  | JF804010 |
| **Gabon=6** | AJ313398 |
|  | AJ313403 |
|  | AJ313405 |
|  | AJ313406 |
|  | AJ313415 |
|  | AJ313418 |
| **Uganda=17** | AF484493 |
|  | AF484507 |
|  | AF484508 |
|  | AF484509 |
|  | AF484512 |
|  | AY713407 |
|  | DQ079832 |
|  | DQ079833 |
|  | DQ079834 |
|  | DQ079835 |
|  | DQ079837 |
|  | DQ079840 |
|  | DQ079841 |
|  | AF000517 |
|  | AF000518 |
|  | AF000519 |
|  | AF177348 |
| Tanzania=16 | AF361872 |
|  | AF361873 |
|  | AY253305 |
|  | AY253314 |
|  | EU251717 |
|  | EU251718 |
|  | EU251722 |
|  | HM572379 |
|  | HM572381 |
|  | HM572387 |
|  | HM572389 |
|  | HM572394 |
|  | JQ071453 |
|  | JQ071454 |
|  | JQ071458 |
|  | JQ730776 |
| Kenya=19 | AF457052 |
|  | AF457053 |
|  | AF457055 |
|  | AF457063 |
|  | AF457065 |
|  | AF457066 |
|  | AF457067 |
|  | AF457068 |
|  | AF457069 |
|  | AF457070 |
|  | AF457075 |
|  | AF457077 |
|  | AF457079 |
|  | AF457080 |
|  | AF457081 |
|  | AF457083 |
|  | AF457084 |
|  | AF457086 |
|  | AF457089 |
| Estonia=9 | GQ926673 |
|  | GQ926676 |
|  | JX431685 |
|  | JX431729 |
|  | JX431732 |
|  | JX431736 |
|  | JX431787 |
|  | [JX431668](http://www.hiv.lanl.gov/components/sequence/HIV/asearch/query_one.comp?se_id=JX431668) |
|  | [JX431670](http://www.hiv.lanl.gov/components/sequence/HIV/asearch/query_one.comp?se_id=JX431670) |
| Latvia=19 | GU945075 |
|  | GU945076 |
|  | GU945077 |
|  | GU945079 |
|  | GU945080 |
|  | GU945081 |
|  | GU945082 |
|  | GU945083 |
|  | GU945084 |
|  | GU945086 |
|  | GU945087 |
|  | GU945088 |
|  | GU945089 |
|  | GU945090 |
|  | GU945091 |
|  | GU945092 |
|  | GU945093 |
|  | GU945094 |
|  | GU945095 |
| Lithuania=5 | JX299690 |
|  | JX299718 |
|  | JX299932 |
|  | JX300086 |
|  | JX300393 |
| Ukraine=20 | DQ055174 |
|  | DQ055175 |
|  | DQ055176 |
|  | DQ055177 |
|  | DQ055178 |
|  | DQ055268 |
|  | DQ055269 |
|  | DQ055270 |
|  | DQ055271 |
|  | DQ055272 |
|  | DQ055297 |
|  | DQ055298 |
|  | DQ055299 |
|  | DQ055301 |
|  | DQ055302 |
|  | DQ055303 |
|  | DQ055304 |
|  | DQ055306 |
|  | DQ055307 |
|  | DQ055308 |
| Moldavia=5 | AF400675 |
|  | AF400676 |
|  | AF400677 |
|  | AF400678 |
|  | AF400679 |
| Byelorussia=10 | EU345608 |
|  | EU345610 |
|  | HE657493 |
|  | HE657516 |
|  | HF679258 |
|  | HF679267 |
|  | HF679260 |
|  | HE657503 |
|  | HE657435 |
|  | HE657490 |
| Russia=20 | [KP659704](http://www.hiv.lanl.gov/components/sequence/HIV/asearch/query_one.comp?se_id=KP659704) |
|  | [KP659705](http://www.hiv.lanl.gov/components/sequence/HIV/asearch/query_one.comp?se_id=KP659705) |
|  | [KP659706](http://www.hiv.lanl.gov/components/sequence/HIV/asearch/query_one.comp?se_id=KP659706) |
|  | [KP659707](http://www.hiv.lanl.gov/components/sequence/HIV/asearch/query_one.comp?se_id=KP659707) |
|  | [KP659708](http://www.hiv.lanl.gov/components/sequence/HIV/asearch/query_one.comp?se_id=KP659708) |
|  | [KP659709](http://www.hiv.lanl.gov/components/sequence/HIV/asearch/query_one.comp?se_id=KP659709) |
|  | [KP659710](http://www.hiv.lanl.gov/components/sequence/HIV/asearch/query_one.comp?se_id=KP659710) |
|  | [KP659711](http://www.hiv.lanl.gov/components/sequence/HIV/asearch/query_one.comp?se_id=KP659711) |
|  | [KP659712](http://www.hiv.lanl.gov/components/sequence/HIV/asearch/query_one.comp?se_id=KP659712) |
|  | [KP659713](http://www.hiv.lanl.gov/components/sequence/HIV/asearch/query_one.comp?se_id=KP659713) |
|  | [KP659714](http://www.hiv.lanl.gov/components/sequence/HIV/asearch/query_one.comp?se_id=KP659714) |
|  | [KP659715](http://www.hiv.lanl.gov/components/sequence/HIV/asearch/query_one.comp?se_id=KP659715) |
|  | [KP659716](http://www.hiv.lanl.gov/components/sequence/HIV/asearch/query_one.comp?se_id=KP659716) |
|  | [KP659717](http://www.hiv.lanl.gov/components/sequence/HIV/asearch/query_one.comp?se_id=KP659717) |
|  | [KP659718](http://www.hiv.lanl.gov/components/sequence/HIV/asearch/query_one.comp?se_id=KP659718) |
|  | [KP659719](http://www.hiv.lanl.gov/components/sequence/HIV/asearch/query_one.comp?se_id=KP659719) |
|  | [KP659720](http://www.hiv.lanl.gov/components/sequence/HIV/asearch/query_one.comp?se_id=KP659720) |
|  | [KP659721](http://www.hiv.lanl.gov/components/sequence/HIV/asearch/query_one.comp?se_id=KP659721) |
|  | [KP659722](http://www.hiv.lanl.gov/components/sequence/HIV/asearch/query_one.comp?se_id=KP659722) |
|  | [KP659723](http://www.hiv.lanl.gov/components/sequence/HIV/asearch/query_one.comp?se_id=KP659723) |
| Kazakhstan=6 | [EF576719](http://www.hiv.lanl.gov/components/sequence/HIV/asearch/query_one.comp?se_id=EF576719) |
|  | [EF576720](http://www.hiv.lanl.gov/components/sequence/HIV/asearch/query_one.comp?se_id=EF576720) |
|  | [EF576721](http://www.hiv.lanl.gov/components/sequence/HIV/asearch/query_one.comp?se_id=EF576721) |
|  | [EF576722](http://www.hiv.lanl.gov/components/sequence/HIV/asearch/query_one.comp?se_id=EF576722) |
|  | [EF576723](http://www.hiv.lanl.gov/components/sequence/HIV/asearch/query_one.comp?se_id=EF576723) |
|  | [EF576724](http://www.hiv.lanl.gov/components/sequence/HIV/asearch/query_one.comp?se_id=EF576724) |
| Uzbekistan=7 | [AY845717](http://www.hiv.lanl.gov/components/sequence/HIV/asearch/query_one.comp?se_id=AY845717) |
|  | [AY845718](http://www.hiv.lanl.gov/components/sequence/HIV/asearch/query_one.comp?se_id=AY845718) |
|  | [AY845719](http://www.hiv.lanl.gov/components/sequence/HIV/asearch/query_one.comp?se_id=AY845719) |
|  | [AY845720](http://www.hiv.lanl.gov/components/sequence/HIV/asearch/query_one.comp?se_id=AY845720) |
|  | [AY845721](http://www.hiv.lanl.gov/components/sequence/HIV/asearch/query_one.comp?se_id=AY845721) |
|  | [AY845722](http://www.hiv.lanl.gov/components/sequence/HIV/asearch/query_one.comp?se_id=AY845722) |
|  | [AY845723](http://www.hiv.lanl.gov/components/sequence/HIV/asearch/query_one.comp?se_id=AY845723) |
| Greece=9 | [AY940271](http://www.hiv.lanl.gov/components/sequence/HIV/asearch/query_one.comp?se_id=AY940271) |
|  | [AY940272](http://www.hiv.lanl.gov/components/sequence/HIV/asearch/query_one.comp?se_id=AY940272) |
|  | [AY940273](http://www.hiv.lanl.gov/components/sequence/HIV/asearch/query_one.comp?se_id=AY940273) |
|  | [AY940274](http://www.hiv.lanl.gov/components/sequence/HIV/asearch/query_one.comp?se_id=AY940274) |
|  | [AY940275](http://www.hiv.lanl.gov/components/sequence/HIV/asearch/query_one.comp?se_id=AY940275) |
|  | [AY940276](http://www.hiv.lanl.gov/components/sequence/HIV/asearch/query_one.comp?se_id=AY940276) |
|  | [AY940277](http://www.hiv.lanl.gov/components/sequence/HIV/asearch/query_one.comp?se_id=AY940277) |
|  | [AY940278](http://www.hiv.lanl.gov/components/sequence/HIV/asearch/query_one.comp?se_id=AY940278) |
|  | [AY940279](http://www.hiv.lanl.gov/components/sequence/HIV/asearch/query_one.comp?se_id=AY940279) |
| Albania=8 | [AY611652](http://www.hiv.lanl.gov/components/sequence/HIV/asearch/query_one.comp?se_id=AY611652) |
|  | [AY611654](http://www.hiv.lanl.gov/components/sequence/HIV/asearch/query_one.comp?se_id=AY611654) |
|  | [AY611655](http://www.hiv.lanl.gov/components/sequence/HIV/asearch/query_one.comp?se_id=AY611655) |
|  | [AY611656](http://www.hiv.lanl.gov/components/sequence/HIV/asearch/query_one.comp?se_id=AY611656) |
|  | [AY611658](http://www.hiv.lanl.gov/components/sequence/HIV/asearch/query_one.comp?se_id=AY611658) |
|  | [AY611659](http://www.hiv.lanl.gov/components/sequence/HIV/asearch/query_one.comp?se_id=AY611659) |
|  | [AY611660](http://www.hiv.lanl.gov/components/sequence/HIV/asearch/query_one.comp?se_id=AY611660) |
|  | [AY611662](http://www.hiv.lanl.gov/components/sequence/HIV/asearch/query_one.comp?se_id=AY611662) |
